# Synthesis and Trypanocidal Activity of Novel 2,4,5-Triaryl-*N*-Hydroxylimidazole Derivatives

**DOI:** 10.3390/molecules18033445

**Published:** 2013-03-15

**Authors:** Ramon Borges da Silva, Vanessa Brandão Loback, Kelly Salomão, Solange Lisboa de Castro, James L. Wardell, Solange M. S. V. Wardell, Thadeu Estevam Moreira Maramaldo Costa, Carmen Penido, Maria das Graças Muller de Oliveira Henriques, Samir Aquino Carvalho, Edson Ferreira da Silva, Carlos Alberto Manssour Fraga

**Affiliations:** 1 Instituto de Tecnologia em Fármacos — Farmanguinhos, Fundação Oswaldo Cruz, 21041-250, Rio de Janeiro, RJ, Brazil; E-Mails: ramonpan@ig.com.br (R.B.S.); nessaloback@gmail.com (V.B.L.); thadeucosta@far.fiocruz.br (T.E.M.M.C.); cpenido@far.fiocruz.br (C.P.); gracahen@far.fiocruz.br (M.G.M.O.H.); scarvalho@far.fiocruz.br (S.A.C.); edsonf@far.fiocruz.br (E.F.S.); 2 Programa de Pós-Graduação em Química, Instituto de Química, Universidade Federal do Rio de Janeiro, 21949-900, Rio de Janeiro, RJ, Brazil; 3 Laboratório de Biologia Celular, Instituto Oswaldo Cruz, Fundação Oswaldo Cruz, 21040-900, Rio de Janeiro, RJ, Brazil; E-Mails: ks@ioc.fiocruz.br (K.S.); solange@ioc.fiocruz.br (S.L.C.); 4 Centro de Desenvolvimento Tecnológico em Saúde (CDTS), Fundação Oswaldo Cruz, 21040-900, Rio de Janeiro, RJ, Brazil; E-Mail: j.wardell@abdn.ac.uk; 5 CHEMSOL, 1 Harcourt Road, Aberdeen AB15 5NY, Scotland, UK; E-Mail: solangewardell@gmail.com; 6 Programa de Pesquisa em Desenvolvimento de Fármacos, Instituto de Ciências Biomédicas, Universidade Federal do Rio de Janeiro, PO Box 68023, 21941-902, Rio de Janeiro, RJ, Brazil

**Keywords:** 2,4,5-triaryl-*N*-hydroxyimidazole, *Trypanosoma cruzi*, Chagas disease, trypanocidal activity

## Abstract

Herein, we report the design, synthesis and trypanocidal activity of some novel trisubstituted imidazole derivatives. These heterocyclic derivatives were structurally planned by exploring the concept of molecular hybridisation between two arylhydrazones derived from megazol, which has potent trypanocidal activity. The trypanocidal activity of these triarylimidazole derivatives was evaluated against infective trypomastigote forms of *T. cruzi* and the derivative 2'-(4-bromophenyl)-1-methyl-5'-phenyl-1*H*,3'*H*-2,4'-biimidazol-3'-ol showed moderate biological activity (IC_50_ = 23.9 µM) when compared to benznidazole, a standard trypanocidal drug. These compounds did not present cytotoxic effects at concentrations near the trypanocidal IC_50_, being considered a good starting point for the development of new anti-Chagas drug candidates.

## 1. Introduction

Chagas disease, also known as American trypanosomiasis or South American trypanosomiasis, is a protozoan disease caused by the haemoflagellate parasite *Trypanosoma cruzi* [1]. It is a chronic and debilitating parasitic infection that affects millions of people in Mexico, Central America, and South America. Approximately 25% of the population of Latin America is at risk for acquiring the infection [2]. Currently, the available drug for the clinical treatment of Chagas disease is the nitroheterocyclic drug benznidazole [3]. This drug is effective against the circulating form of the parasite (trypomastigotes) in the acute phase of the disease, but its efficacy during the chronic stage is debatable [4].

Megazol [1-methyl-2-(5-amino-1,3,4-thiadiazole)-5-nitroimidazole] is a nitroheterocyclic derivative shown to be highly active against *T. cruzi in vitro* and *in vivo*, including strains that are resistant to benznidazole [5,6,7]; thus, it has become a core structure for the design of new drugs for the treatment of Chagas disease. Megazol has been described as a scavenger of trypanothione, the cofactor for trypanothione reductase [8,9]. Despite its noteworthy trypanocidal activity, megazol development was discontinued due to reports of its *in vitro* mutagenic and genotoxic effects [10,11,12]. To circumvent this undesirable profile, there have been numerous efforts to obtain megazol analogues [13,14,15,16,17].

The imidazole ring is commonly found in highly significant endogenous biomolecules including biotin, the essential amino acid histidine and the autacoid histamine [18]. Several bioactive compounds with this heterocyclic unit have valuable pharmacological properties such as antiparasitic [19], antifungal [20], antimicrobial [21,22] and antidepressant [23] activity, among others. In this context, 2,4,5-triarylimidazole compounds have gained remarkable importance due to their widespread biological activities and their applicability in synthetic organic chemistry. Moreover, *N*-hydroxyimidazoles have been reported to possess fungicidal and bacteriostatic activities [24].

**Figure 1:**
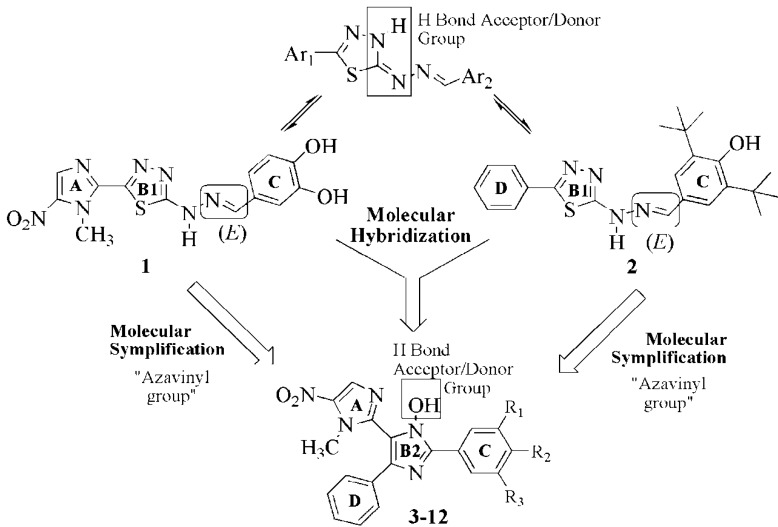
Design concept of the new triaryl-*N*-hydroxyimidazole derivatives **3**–**12**.

In our continuous effort to develop potent trypanocidal compounds, we decided to construct a new class of 2,4,5-triaryl-*N*-hydroxyimidazole (TAI) derivatives **3**–**12** based on the molecular hybridisation [25] of 1,3,4-thiadiazole prototypes **1** and **2** (Figure 1) [14]. In the design concept, the nitroimidazole moiety (A) was preserved due to the pharmacophoric contribution of this group to the mechanism of action against *T. cruzi* [14]. The 1,3,4-thiadiazole group (B1) of **1** and **2** was isosterically substituted by an imidazole ring (B2) containing a hydroxyl group that mimics the proton donor/accepting behaviour of the tautomeric N-H bond of the hydrazone group attached to B1 (Figure 1).

## 2. Results and Discussion

### 2.1. Chemistry

**Scheme 1:**
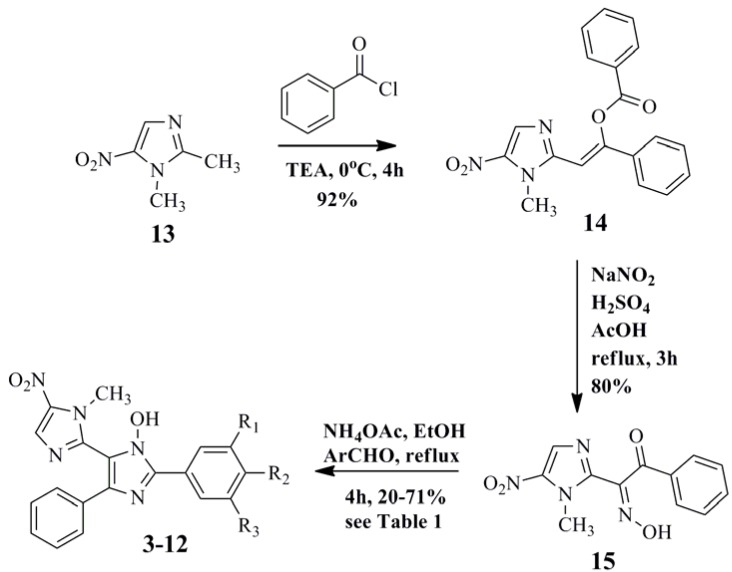
Synthesis of the new triaryl-*N*-hydroxyimidazole derivatives **3**–**12**

**Figure 2:**
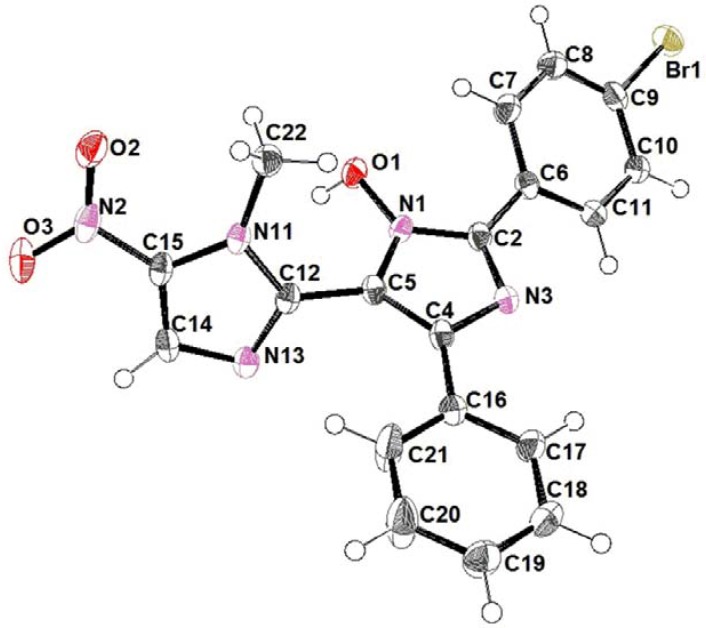
Atom arrangements for 2'-(4-bromophenyl)-1-methyl-5-nitro-5'-phenyl-1*N*,3'*N*-2,4'-biimidazol-3'-ol (**6**).

The synthetic route used for the preparation of the title compounds **3**–**12** is outlined in Scheme 1. 1,2-Dimethyl-5-nitro-1*H*-imidazole (**13**) was converted into the corresponding phenylvinyl benzoate **14** in 92% yield through its base-catalysed condensation with benzoyl chloride [26]. Next, the nitrosation of **14** furnished the key ketoxime intermediate **15** in 80% yield [26]. Finally, the desired TAI derivatives **3**–**12** were obtained, in yields varying from 20–70%, after condensation of **15** with the corresponding benzaldehydes in the presence of ammonium acetate [27]. The ^1^H- and ^13^C-NMR spectra and mass spectra of the synthesised compounds **3**–**12** were consistent with the proposed structures, which was corroborated by X-ray crystallography of the *p*-bromo-TAI derivative **6** (CCDC 928948 contains the supplementary crystallographic data for this paper. These data can be obtained free of charge via www.ccdc.cam.ac.uk/conts/retrieving.html) as illustrated in Figure 2.

### 2.2. Trypanocidal Activity

The trypanocide profiles of the new TAI derivatives **3**–**12** was evaluated *in vitro* using bloodstream trypomastigote forms of *T. cruzi* (Y strain) isolated from infected Swiss mice and are summarised in Table 1.

**Table 1:** Physical and biological properties of 2,4,5-triaryl-*N*-hydroxyimidazole derivatives **3**–**12**. 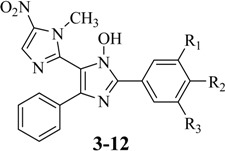

Cpn	R_1_	R_2_	R_3_	Molecular Formula	M.W.	Yield (%)	M.P. (°C)	IC_50_ (µM) ^a^	log P values ^b^
3	H	H	H	C_19_H_15_N_5_O_3_	361.35	69	217–218	108.2 ± 5.65	2.92 ± 0.98
4	H	F	H	C_19_H_14_FN_5_O_3_	379.38	48	248–249	67.8 ± 8.39	3.14 ± 1.02
5	H	Cl	H	C_19_H_14_ClN_5_O_3_	395.80	20	249–250	34.6 ± 2.43	3.69 ± 0.99
6	H	Br	H	C_19_H_14_BrN_5_O_3_	440.25	69	218–219	23.9 ± 4.88	3.86 ± 1.02
7	H	NO_2_	H	C_19_H_14_N_6_O_5_	406.35	62	361–362	241.6 ± 37.6	2.88 ± 0.99
8	H	OCH_3_	H	C_20_H_17_N_5_O_4_	391.38	62	218–219	191.8 ± 25.3	3.09 ± 0.99
9	H	OH	H	C_19_H_15_N_5_O_4_	377.35	71	145–146	294.1 ± 27.98	2.56 ± 0.99
10	OH	OH	H	C_20_H_15_N_5_O_5_	393.35	42	224–225	360.5 ± 24.67	2.34 ± 0.99
11	OH	OCH_3_	H	C_20_H_17_FN_5_O_5_	407.38	45	213–214	199.9 ± 2.02	2.53 ± 1.00
12	OCH_3_	OH	NO_2_	C_20_H_16_N_6_O_7_	452.38	43	245–247	241.8 ± 7.54	3.22 ± 1.01
Bzn	-	-	-	-	-	-	-	10.8 ± 0.4	0.91 ± 1.00

^a^ Mean ± standard deviation of at least four separate experiments, performed with trypomastigote forms of *T. cruzi*; ^b^ Theoretical values calculated using the program ACDLABS.

**Figure 3:**
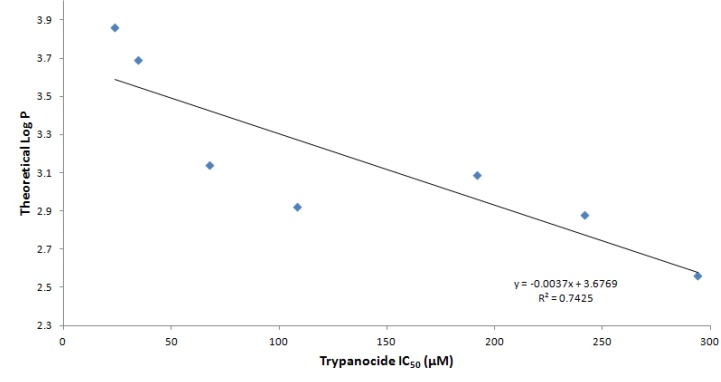
Correlation between the theoretical log P value of TAI derivatives **3**–**9** and their corresponding trypanocidal activity (see Table 1).

The screening of the TAI derivatives **3**–**12** showed that compound **6**, with a *p*-bromophenyl group attached to the core imidazole ring, was the most active, with an IC_50_ = 23.9 µM, which is slightly inferior to that displayed by the standard drug benznidazole (IC_50_ = 10.8 µM). The superior trypanocidal profile of **6**, compared with the other monosubstituted TAI analogues **3**–**5** and **7**–**9**, could be explained by the remarkable hydrophobic contribution of its bromo group (Hantzsch’s π = 0.86), increasing the log P of this compound to 3.86 (Table 1). We did not find a clear correlation among the stereoelectronic character of *para*-substituents in TAI compounds **3**–**9** and their trypanocidal activity. In fact, the introduction of *para*-substituents from different halogens, independently of any electron-withdrawing or electron-releasing properties, prominently reduced the trypanocidal profile when compared with an unsubstituted TAI derivative **3**. Nevertheless, we have found a good correlation (r^2^ = 0.74) between theoretical log P value and the trypanocide profile of monosubstituted TAI derivatives **3**–**9**, indicating the importance of their relative lipophilicity for the displayed biological activity (Figure 3).

Di- and tri-substituted TAI derivatives **10**–**12** have shown poor trypanocidal activity, distinct from the corresponding brazilizone derivatives, e.g., **1** [14,15].

The cellular viability in the presence of most active TAI derivatives **4**–**6** was determined by microplate Alamar Blue assay (Invitrogen) at three different concentrations, 100, 10 and 1 μM. The results displayed in Table 2 were expressed in percentage cell viability. Excepting the *p*-bromo TAI derivative **6**, that weakly reduced cell viability at 100 μM, the other tested compounds were not cytotoxic to the host cells at concentrations near the IC_50_ for trypanocidal action (Table 1). 

**Table 2:** Data of cytotoxic effects of 2,4,5-triaryl-*N*-hydroxyimidazole derivatives (**4**–**6**) on murine macrophages cells 21 h after the treatment.

TAI Compound	% Cell Viability/concentration (μM)		
	100	10	1
4	97.6	98.9	94.2
5	93.1	97.6	95.7
6	78.7	97.6	97.3

## 3. Experimental

### 3.1. General Procedures

Melting points were determined on a Buchi apparatus and are uncorrected. Infrared spectra were recorded on a Thermo Nicolet Nexus 670 spectrometer in potassium bromide pellets and frequencies are expressed in cm^−1^. ^1^H-NMR spectra were recorded at room temperature on Bruker Avance 500 and Bruker Avance 400 spectrometers operating at 500/125 and 400/100 MHz (^1^H/^13^C), respectively. Chemical shifts are reported in ppm (δ) downfield from tetramethylsilane, which was used as an internal standard. Low resolution mass spectra (MS) were obtained by electron-spray ionisation in a micromass ZQ 4000. Microanalysis data were obtained using a Perkin–Elmer 240 analyser, using a Perkin–Elmer AD-4 balance. The progress of all reactions was monitored by TLC, which was performed on 2.0 X 6.0 cm aluminium sheets that were precoated with silica gel 60 (HF-254, Merck) to a thickness of 0.25 mm. The developed chromatograms were viewed under ultraviolet light (254–265 nm).

### 3.2. Procedure for the Synthesis of (Z)-2-(1-Methyl-5-nitro-1H-imidazol-2-yl)-1-phenylvinyl benzoate (**14**) [26]

In a 250 mL flask, cooled in an ice bath, acetone (34 mL) and 1,2-dimethyl-5-nitroimidazole (**13**, 10 g, 70.85 mmol) were added while stirring. Next, triethylamine (50 mL, 70.85 mmol) and then benzoyl chloride (24.4 mL, 210.0 mmol, 3 equiv.) were added slowly. The temperature was adjusted to 20 °C, and acetone (46 mL) was then added. The reaction was stirred for 4 hours. Next, water (40 mL) was added, and the suspension was filtered on a Buchner funnel and washed with acetone, affording 25 g (92% yield) of **14**. Light green solid; mp 245–275 °C; ^1^H-NMR (500 MHz, DMSO-d_6_) δ 4.05 (s, 3H, 5-NO_2_-imidazole-*N*-CH_3_), 7.28 (s, 1H, 5-NO_2_-imidazole-CH=C-), 7.49 (m, 3H, Ar(C_3_)H, Ar(C_4_)H and Ar(C_5_)H), 7.61 (t, 2H, benzoate(C_3_)H and benzoate(C_5_)H), 7.75 (t, 1H, benzoate(C_4_)H), 7.83 (m, 2H, Ar(C_2_)H and Ar(C_6_)H), 7.99 (s, 1H, 5-NO_2_-imidazole-(C_4_)H), 8.13 (d, 2H, benzoate(C_2_)H and benzoate(C_6_)H): ^13^C-NMR (125 MHz, DMSO-d_6_) δ 33.16 (5-NO_2_-imidazole-*N*-CH_3_), 101.90 (5-NO_2_-imidazole-(C_2_)-CH=C-), 125.39 (benzoate(C_3_)H and benzoate(C_5_)H), 128.49 (benzoate(C_1_)), 128.97 (Ar(C_3_)H and Ar(C_5_)H), 130.00 (Ar(C_2_)H and Ar(C_6_)H), 130.29 (benzoate(C_2_)H and benzoate(C_6_)H), 132.59 (Ar(C_1_)), 133.74 (5-NO_2_-imidazole-(C_4_)H and Ar(C_4_)H), 133.90 benzoate-(C_4_)H), 138.76 (5-NO_2_-imidazole-(C_2_)CH=C-), 146.44 (5-NO_2_-imidazole-(C_5_)NO_2_), 152.24 (5-NO_2_-imidazole-(C_2_)CH=C-), 163.71(Ar-COO-),: MS (ESI) *m/z*: 372.0 (M^+.^[+Na]) (100%). Anal. Calcd. for C_19_H_15_N_3_O_4_: C: 65.32; H: 4.33; N: 12.03. Found: C: 65.31; H: 4.33; N: 12.03.

### 3.3. Procedure for the Synthesis of (1E)-1-(1-Methyl-5-nitro-1H-imidazol-2-yl)-2-phenylethane-1,2-dione 1-oxime (**15**) [26]

In small portions, NaNO_2_ (3.82 g, 55.36 mmol) were added to a 250 mL flask that was seeded with H_2_SO_4_ (56.6 mL) and cooled in an ice bath while stirring. After 30 min, glacial acetic acid (20.6 mL) and then phenylvinyl benzoate **14** (10.0 g, 28.62 mmol) were added. The reaction was maintained at 65 °C for 3 h and the temperature was then adjusted to 20 °C. Next, water (166 mL) was added, and this mixture was subsequently filtered in a Buchner funnel. Compound **15** was suspended in ethanol and filtered through a Buchner funnel, resulting in a yellow solid with a yield of 80%. mp 180–182 °C; ^1^H-NMR (500 MHz, DMSO-d_6_) δ 3.78 (s, 3H, 5-NO_2_-imidazole-*N*-CH_3_), 7.58 (m, 2H, Ar(C_3_)H and Ar(C_5_)H), 7.71 (t, 1H, Ar(C_4_)H), 7.98 (m, 2H, Ar(C_2_)H and Ar(C_6_)H), 8.21 (s, 1H, 5-NO_2_-imidazole-(C_4_)H), 13.85 (s, 1H, C=N-OH): ^13^C-NMR (125 MHz, DMSO-d_6_) δ 34.51 (5-NO_2_-imidazole-*N*-CH_3_), 128.47 (Ar(C_3_)H and Ar(C_5_)H), 130.16 (Ar(C_2_)H and Ar(C_6_)H), 132.24 (5-NO_2_-imidazole-(C_4_)H), 133.53 (Ar(C_4_)H), 135.79 (5-NO_2_-imidazole-(C_5_)NO_2_), 139.45 (5-NO_2_-imidazole-(C_2_)-C=N-OH), 142.46 (Ar(C_1_)), 146.04 (C=N-OH), 189.07 (C=O): MS (ESI) *m/z*: 273.1 (100%). Anal. Calcd. for C_12_H_10_N_4_O_4_: C: 52.56; H: 3.68; N: 20.43. Found: C: 52.54; H: 3.68; N: 20.42.

### 3.4. General Procedure for the Synthesis of 2,4,5-Trisubstituted Imidazole Derivatives (**3**–**12**) [27]

In a 100 mL flask, ketoxime **15** (0.5 g, 1.82 mmol, 1 eq.), NH_4_OAc (0.850 g, 11.02 mmol, 6 eq.), the corresponding benzaldehyde (1.82 mmol, 1 eq.) and ethanol (12 mL) were added while stirring. The temperature was maintained at reflux for 5 h. The reaction was cooled 20 °C and then placed on crushed ice. The precipitate was filtered on a Buchner funnel and washed with water. All new 2,4,5-triaryl-*N*-hydroxyimidazole derivatives **3**–**12** were purified by recrystallization from a 9:1 solution of ethanol/water.

*1-Methyl-5-nitro-2',5'-diphenyl-1H,3'H-2,4'-biimidazol-3'-ol* (**3**). Yellow solid; mp 217–218 °C; yield 69%; ^1^H-NMR (500 MHz, DMSO-d_6_) δ 3.72 (5-NO_2_-imidazole-*N*-CH_3_), 7.28 (t, 1H, Ar(C_4_)H), 7.34 (t, 2H, Ar(C_3_)H and Ar(C_5_)H), 7.47 (t, 1H, Ar(C_4’_)H), 7.54 (t, 2H, Ar(C_3’_)H and Ar(C_5’_)H), 7.57 (d, 2H, Ar(C_2_)H and Ar(C_6_)H), 8.16 (d, 2H, Ar(C_2’_)H and Ar(C_6’_)H), 8.35 (s, 1H, 5-NO_2_-imidazole-(C_4_)H), 12.48 (s, 1H, N-OH-imidazole-*N*-OH): ^13^C-NMR (125 MHz, DMSO-d_6_) δ 34.57 (5-NO_2_-imidazole-*N*-CH_3_), 115.87 (*N*-OH-imidazole-C_4_), 125.89 (Ar(C_2_)H and Ar(C_6_)H), 127.39 (Ar(C_2’_)H and Ar(C_6’_)H), 127.71 (Ar(C_4_)H), 128.42 (Ar(C_1’_)), 128.61 (Ar(C_3_)H and Ar(C_5_)H), 128.70 (Ar(C_3’_)H and Ar(C_5’_)H), 129.33 (Ar(C_4’_)H), 132.75 (5-NO_2_-imidazole-(C_4_)H), 133.10 (*N*-OH-imidazole-C_2_), 137.56 (Ar(C_1_)), 139.97 (5-NO_2_-imidazole-(C_2_)), 141.28 (5-NO_2_-imidazole-(C_5_)NO_2_), 142.25 (*N*-OH-imidazole-C_5_): ^13^C-NMR DEPT (100 MHz, DMSO-d_6_)δ 34.57 (5-NO_2_-imidazole-*N*-CH_3_), 125.89 (Ar(C_2_)H and Ar(C_6_)H), 127.39 (Ar(C_2’_)H and Ar(C_6’_)H), 127.71 (Ar(C_4_)H), 128.61 (Ar(C_3_)H and Ar(C_5_)H), 128.70 (Ar(C_3’_)H and Ar(C_5’_)H), 129.33 (Ar(C_4’_)H), 132.75 (5-NO_2_-imidazole-(C_4_)H): IR (KBr) ν_max_ cm^−1^: 3284 (ν *N*-O-H), 3130–3064 (ν C-H(aromatic), 1526–1470 (ν C=C(aromatic), 1368 (ν N=O_2_): MS (ESI) *m/z*: 360.5 (100%). Anal. Calcd. for C_19_H_15_N_5_O_3_: C: 63.15; H: 4.18; N: 19.38. Found: C: 63.17; H: 4.18; N: 19.40.

*2'-(4-Fluorophenyl)-1-methyl-5-nitro-5'-phenyl-1H,3'H-2,4'-biimidazol-3'-ol* (**4**). Yellow solid; mp 148–149 °C; yield 48%; ^1^H-NMR (400 MHz, DMSO-d_6_) δ 3.87 (5-NO_2_-imidazole-*N*-CH_3_), 7.27 (t, 1H, Ar(C_4_)H), 7.34 (t, 2H, Ar(C_3’_)H and Ar(C_5’_)H), 7.39 (d, 2H, Ar(C_2’_)H and Ar(C_6’_)H), 7.56 (d, 2H, Ar(C_2_)H and Ar(C_6_)H), 8.19 (t, 2H, Ar(C_3’_)H and Ar(C_5’_)H), 8.34 (s, 1H, 5-NO_2_-imidazole-(C_4_)H), 12.49 (s, 1H, *N*-OH-imidazole-*N*-OH): ^13^C-NMR (100 MHz, DMSO-d_6_) δ 34.51 (5-NO_2_-imidazole-*N*-CH_3_), 115.72 (d, *J* = 21.8 Hz, Ar(C_3’_)H and Ar(C_5’_)H), 124.95 (*N*-OH-imidazole-(C_4_)), 125.85 (Ar(C_2_)H and Ar(C_6_)H), 127.70 (Ar(C_4_)H), 128.57 (Ar(C_3_)H and Ar(C_5_)H), 129.63 (d, *J* = 8.7 Hz, Ar(C_2’_)H and Ar(C_6’_)H), 132.70 (5-NO_2_-imidazole-(C_4_)H), 132.97 (*N*-OH-imidazole-(C_2_)), 137.49 (Ar(C_1_)), 139.95 (5-NO_2_-imidazole-(C_2_)), 141.17 (5-NO_2_-imidazole-(C_5_)NO_2_), 141.42 (*N*-OH-imidazole-(C_5_)), 162.52 (d, *J* = 245.8 Hz, Ar(C_4’_)F): IR (KBr) ν_max_ cm^−1^: 3284 (ν *N*-O-H), 3130–3066 (ν C-H(aromatic), 1534–1468 (ν C=C(aromatic), 1365 (ν N=O_2_), 1225 (ν C-F): MS (ESI) *m/z*: 378.2 (100%). Anal. Calcd. for C_19_H_14_FN_5_O_3_: C: 60.16; H: 3.72; N: 18.46. Found: C: 60.18; H: 3.72; N: 18.47.

*2'-(4-Chlorophenyl)-1-methyl-5-nitro-5'-phenyl-1H,3'H-2,4'-biimidazol-3'-ol* (**5**). green solid; mp 149–250 °C; yield 20%; ^1^H-NMR (500 MHz, DMSO-d_6_) δ 3.71 (5-NO_2_-imidazole-N-CH_3_), 7.27 (t, 1H, Ar(C_4_)H), 7.33 (t, 2H, Ar(C_3_)H and Ar(C_5_)H), 7.55 (d, 2H, *J* = 7.5 Hz, Ar(C_2_)H and Ar(C_6_)H), 7.60 (d, 2H, *J* = 7.5 Hz, Ar(C_2’_)H and Ar(C_6’_)H), 8.17 (d, 2H, *J* = 7.5 Hz, Ar(C_3’_)H and Ar(C_5’_)H), 8.33 (s, 1H, 5-NO_2_-imidazole-(C_4_)H), 12.58 (s, 1H, *N*-OH-imidazole-*N*-OH): ^13^C-NMR (125 MHz, DMSO-d_6_) δ 34.54 (5-NO_2_-imidazole-*N*-CH_3_), 116.14 (*N*-OH-imidazole-(C_4_)), 125.86 (Ar(C_2_)H and Ar(C_6_)H), 127.72 (Ar(C_4_)H), 128.58 (Ar(C_3_)H and Ar(C_5_)H), 128.77 (Ar(C_2’_)H and Ar(C_6’_)H), 128.86 (Ar(C_3’_)H and Ar(C_5’_)H), 132.72 (5-NO_2_-imidazole-(C_4_)H), 133.86 (*N*-OH-imidazole-(C_2_)), 137.60 (Ar(C_1_)), 139.94 (5-NO_2_-imidazole-(C_2_)), 141.10 (5-NO_2_-imidazole-(C_5_)NO_2_): IR (KBr) ν_max_ cm^−1^: 3437 (ν *N*-O-H), 3144–3058 (ν C-H(aromatic), 1533–1470 (ν C=C(aromatic), 1364 (ν N=O_2_): MS (ESI) *m/z*: 394.5 (100%). Anal. Calcd. for C_19_H_14_ClN_5_O_3_: C: 57.66; H: 3.57; N: 17.69. Found: C: 57.68; H: 3.57; N: 17.69.

*2'-(4-Bromophenyl)-1-methyl-5-nitro-5'-phenyl-1H,3'H-2,4'-biimidazol-3'-ol* (**6**). Yellow solid; mp 218–219 °C; yield 69%; ^1^H-NMR (500 MHz, DMSO-d_6_) δ 3.71 (s, 3H, 5-NO_2_-imidazole-*N*-CH_3_), 7.28 (t, 1H, Ar(C_4_)H), 7.33 (t, 2H, Ar(C_3_)H and Ar(C_5_)H), 7.55 (d, 2H, *J* = 8.0 Hz, Ar(C_2_)H and Ar(C_6_)H), 7.75 (d, 2H, *J* = 8.0 Hz, Ar(C_2’_)H and Ar(C_6’_)H), 8.10 (d, 2H, *J* = 8.0 Hz, Ar(C_3’_)H and Ar(C_5’_)H), 8.34 (s, 1H, 5-NO_2_-imidazole-(C_4_)H), 12.51 (s, 1H, *N*-OH-imidazole-N-OH): ^13^C-NMR (125 MHz, DMSO-d_6_) δ 34.54 (5-NO_2_-imidazole-*N*-CH_3_), 116.15 (*N*-OH-imidazole-(C_4_)), 122.68 (Ar(C_4’_)Br), 125.84 (Ar(C_2_)H and Ar(C_6_)H), 127.54 (Ar(C_1’_)), 127.75 (Ar(C_4_)H), 128.60 (Ar(C_3_)H and Ar(C_5_)H), 129.12 (Ar(C_2’_)H and Ar(C_6’_)H), 131.73 (Ar(C_3’_)H and Ar(C_5’_)H), 132.71 (5-NO_2_-imidazole-(C_4_)H), 132.89 (*N*-OH-imidazole-(C_2_)), 137.66 (Ar(C_1_)), 139.97 (5-NO_2_-imidazole-(C_2_)), 141.00 (5-NO_2_-imidazole-(C_5_)NO_2_), 141.15 (*N*-OH-imidazole-(C_5_)): IR (KBr) ν_max_ cm^−1^: 3140–3056 (ν C-H(aromatic), 1533–1470 (ν C=C(aromatic), 1364 (ν N=O_2_), 828 (ν C-N (Ar-NO_2_),: MS (ESI) *m/z*: 440.3 (100%). Anal. Calcd. for C_19_H_14_BrN_5_O_3_: C: 51.83; H: 3.21; N: 15.91. Found: C: 51.85; H: 3.21; N: 15.91.

*1-Methyl-5-nitro-2'-(4-nitrophenyl)-5'-phenyl-1H,3'H-2,4'-biimidazol-3'-ol* (**7**). Yellow solid; mp 361–362 °C; yield 62%; ^1^H-NMR (500 MHz, DMSO-d_6_) δ 3.73 (s, 3H, 5-NO_2_-imidazole-N-CH_3_), 7.27 (t, 1H, Ar(C_4_)H), 7.33 (t, 2H, Ar(C_3_)H and Ar(C_5_)H), 7.57 (d, 2H, Ar(C_2_)H and Ar(C_6_)H), 7.80 (d, 1H, Ar(C_2’_)H, Ar(C_3’_)H, Ar(C_5’_)H, or Ar(C_6’_)H), 7.92 (t, 1H, Ar(C_2’_)H, Ar(C_3’_)H, Ar(C_5’_)H, or Ar(C_6’_)H), 7.97 (t, 1H, Ar(C_2’_)H, Ar(C_3’_)H, Ar(C_5’_)H, or Ar(C_6’_)H), 8.15(d, 1H, Ar(C_2’_)H, Ar(C_3’_)H, Ar(C_5’_)H, or Ar(C_6’_)H), 8.33 (s, 1H, 5-NO_2_-imidazole-(C_4_)H), 12.40 (s, 1H, *N*-OH-imidazole-*N*-OH): ^13^C-NMR (125 MHz, DMSO-d_6_) δ 34.39 (5-NO_2_-imidazole-*N*-CH_3_), 115.50 (*N*-OH-imidazole-(C_4_)), 124.63 (Ar(C_3’_)H and Ar(C_5’_)H), 125.94 (Ar(C_2_)H and Ar(C_6_)H), 127.78 (Ar(C_4_)H), 128.56 (Ar(C_3_)H and Ar(C_5_)H), 131.15 (Ar(C_2’_)H and Ar(C_6’_)H), 132.60 (5-NO_2_-imidazole-(C_4_)H), 132.79 (*N*-OH-imidazole-(C_2_)), 133.64 (Ar(C_1_)), 139.49 (*N*-OH-imidazole-(C_5_)), 140.05 (5-NO_2_-imidazole-(C_2_)NO_2_), 140.72 (5-NO_2_-imidazole-(C_5_)NO_2_), 148.48 (Ar(C_4’_)NO_2_): IR (KBr) ν_max_ cm^−1^: 3120–3033 (ν C-H(aromatic), 1524–1465 (ν C=C(aromatic), 1349–1365 (ν N=O_2_), 826 (ν C-N (Ar-NO_2_),: MS (ESI) *m/z*: 405.3 (100%). Anal. Calcd. for C_19_H_14_N_6_O_5_: C: 56.16; H: 3.47; N: 20.68. Found: C: 56.15; H: 3.47; N: 20.67.

*2'-(4-Methoxyphenyl)-1-methyl-5-nitro-5'-phenyl-1H,3'H-2,4'-biimidazol-3'-ol* (**8**). Yellow solid; mp 218–219 °C; yield 62%; ^1^H-NMR (400 MHz, DMSO-d_6_) δ 3.73 (s, 3H, 5-NO_2_-imidazole-N-CH_3_), 3.81 (s, 3H, Ar(C_4’_)OCH_3_), 7.08 (t, 1H, *J* = 8.4 Hz, Ar(C_2’_)H, Ar(C_3’_)H, Ar(C_5’_)H or Ar(C_6’_)H), 7.17 (d, 1H, *J* = 8.4 Hz, Ar(C_2’_)H, Ar(C_3’_)H, Ar(C_5’_)H or Ar(C_6’_)H), 7.25 (t, 1H, Ar(C_4_)H), 7.32 (t, 2H, Ar(C_2_)H, Ar(C_3_)H, Ar(C_5_)H or Ar(C_6_)H), 7.51 (t, 2H, Ar(C_3’_)H and Ar(C_5’_)H), 7.56 (d, 2H, *J* = 7.2 Hz, Ar(C_2_)H and Ar(C_6_)H), 8.34 (s, 1H, 5-NO_2_-imidazole-(C_4_)H), 11.72 (s, 1H, *N*-OH-imidazole-N-OH): ^13^C-NMR (100 MHz, DMSO-d_6_) δ 34.43 (5-NO_2_-imidazole-*N*-CH_3_), 55.72 (Ar(C_4’_)OCH_3_), 111.77 and 120.18 (Ar(C_3’_)H and Ar(C_5’_)H), 114.71 (*N*-OH-imidazole-(C_4_)), 117.77 (Ar(C_1’_)), 125.82 (Ar(C_2_)H and Ar(C_6_)H), 127.38 (Ar(C_4_)H), 128.43 (Ar(C_3_)H and Ar(C_5_)H), 131.37 and 131.54 (Ar(C_2’_)H and Ar(C_6’_)H), 132.63 (5-NO_2_-imidazole-(C_4_)H), 133.35 (*N*-OH-imidazole-(C_2_)), 137.48 (Ar(C_1_)), 139.85 (5-NO_2_-imidazole-(C_2_)), 141.58 (5-NO_2_-imidazole-(C_5_)NO_2_), 141.76 (*N*-OH-imidazole-(C_5_)), 157.70 (Ar(C_4’_)OCH_3_): IR (KBr) ν_max_ cm^−1^: 3448 (ν *N*-O-H), 3070–3054 (ν C-H(aromatic), 1471–1488 (ν C=C(aromatic), 1363 (ν N=O_2_), 825 (ν C-N (Ar-NO_2_),: MS (ESI) *m/z*: 390.4 (100%). Anal. Calcd. for C_20_H_17_N_5_O_4_: C: 56.16; H: 3.47; N: 20.68. Found: C: 56.15; H: 3.47; N: 20.67.

*2'-(4-Hydroxyphenyl)-1-methyl-5-nitro-5'-phenyl-1H,3'H-2,4'-biimidazol-3'-ol* (**9**). Yellow solid; mp 145–146 °C; yield 71%; ^1^H-NMR (400 MHz, DMSO-d_6_) δ 3.70 (s, 3H, 5-NO_2_-imidazole-*N*-CH_3_), 6.90 (d, 2H, *J* = 9.2 Hz, Ar(C_3’_)H and Ar(C_5’_)H), 7.25 (t, 1H, Ar(C_4_)H), 7.32 (t, 2H, Ar(C_3_)H and Ar(C_5_)H), 7.53 (d, 2H, *J* = 7.6 Hz, Ar(C_2_)H and Ar(C_6_)H), 7.97 (d, 2H, *J* = 8.4 Hz, Ar(C_2’_)H and Ar(C_6’_)H), 8.36 (s, 1H, 5-NO_2_-imidazole-(C_4_)H), 9.89 (s, 1H, Ar(C_4’_)OH), 12.21 (s, 1H, *N*-OH-imidazole-N-OH): ^13^C-NMR (100 MHz, DMSO-d_6_) δ 34.52 (5-NO_2_-imidazole-*N*-CH_3_), 115.09 (*N*-OH-imidazole-(C_4_)), 115.42 (Ar(C_3’_)H and Ar(C_5’_)H), 119.40 (Ar(C_1’_)), 125.79 (Ar(C_2_)H and Ar(C_6_)H), 127.51 (Ar(C_4_)H), 128.54 (Ar(C_3_)H and Ar(C_5_)H), 129.04 (Ar(C_2’_)H and Ar(C_6’_)H), 132.75 (5-NO_2_-imidazole-(C_4_)H), 133.26 (*N*-OH-imidazole-(C_2_)), 137.13 (Ar(C_1_)), 139.87 (5-NO_2_-imidazole-(C_2_)), 141.56 (5-NO_2_-imidazole-(C_5_)NO_2_), 142.74 (*N*-OH-imidazole-(C_5_)), 158.50 (Ar(C_4’_)OH): IR (KBr) ν_max_ cm^−1^: 3123–3062 (ν C-H(aromatic), 1527–1465 (ν C=C(aromatic), 1283 (ν N=O_2_), 827 (ν C-N (Ar-NO_2_),: MS (ESI) *m/z*: 376.3 (100%). Anal. Calcd. for C_19_H_15_N_5_O_4_: C: 60.47; H: 4.01; N: 18.56. Found: C: 60.49; H: 4.01; N: 18.56.

*4-(3'-Hydroxy-1-methyl-5-nitro-5'-phenyl-1H,3'H-2,4'-biimidazol-2'-yl)benzene-1,2-diol* (**10**). Yellow solid; mp 224–225 °C; yield 42%; ^1^H-NMR (500 MHz, DMSO-d_6_) δ 3.70 (s, 3H, 5-NO_2_-imidazole-*N*-CH_3_), 6.85 (d, 1H, *J* = 8.0 Hz, Ar(C_5’_)H), 7.25 (t, 1H, Ar(C_4_)H), 7.32 (t, 2H, Ar(C_3_)H and Ar(C_5_)H), 7.49 (dd, 1H, *J* = 8.5 Hz, Ar(C_6’_)H), 7.53 (d, 2H, Ar(C_2_)H and Ar(C_6_)H), 7.62 (d, 1H, Ar(C_2’_)H), 8.32 (s, 1H, 5-NO_2_-imidazole-(C_4_)H), 9.23 (s, 1H, Ar(C_3’_)OH or Ar(C_4’_)OH), 9.33 (s, 1H, Ar(C_3’_)OH or Ar(C_4’_)OH), 12.16 (s, 1H, *N*-OH- midazole-*N*-OH): ^13^C-NMR (125 MHz, DMSO-d_6_) δ 34.54 (5-NO_2_-imidazole-N-CH_3_), 114.87 (Ar(C_2’_)H), 115.09 (*N*-OH-imidazole-(C_4_)), 115.63 (Ar(C_5’_)H), 119.17 (Ar(C_6’_)H), 119.69 (Ar(C_1’_)), 125.80 (Ar(C_2_)H and Ar(C_6_)H), 127.49 (Ar(C_4_)H), 128.54 (Ar(C_3_)H and Ar(C_5_)H), 132.77 (5-NO_2_-imidazole-(C_4_)H), 133.31 (*N*-OH-imidazole-(C_2_)), 137.05 (Ar(C_1_)), 139.86 (5-NO_2_-imidazole-(C_2_)), 141.61 (5-NO_2_-imidazole-(C_5_)NO_2_), 142.69 (*N*-OH-imidazole-(C_5_)), 145.20 Ar(C_3’_)OH), 146.82 Ar(C_4’_)OH): IR (KBr) ν_max_ cm^−1^: 3367 (ν O-H), 3124–3066 (ν C-H(aromatic), 1528–1468 (ν C=C(aromatic), 1365 (ν N=O_2_), 827 (ν C-N (Ar-NO_2_),: MS (ESI) *m/z*: 392.2 (100%). Anal. Calcd. for C_19_H_15_N_5_O_5_: C: 58.01; H: 3.84; N: 17.80. Found: C: 57.09; H: 3.84; N: 17.79.

*2'-(3-Hydroxy-4-methoxyphenyl)-1-methyl-5-nitro-5'-phenyl-1H,3'H-2,4'-biimidazol-3'-ol* (**11**). Gray solid; mp 213 °C; yield 45%; ^1^H-NMR (400 MHz, DMSO-d_6_) δ 3.70 (s, 3H, 5-NO_2_-imidazole-*N*-CH_3_), 3.82 (s, 3H, Ar(C_4’_)OCH_3_), 7.05 (d, 1H, Ar(C_2_)H, Ar(C_5_)H or Ar(C_6_)H), 7.26 (t, 1H, Ar(C_4_)H), 7.33 (t, 2H, Ar(C_3_)H and Ar(C_5_)H), 7.53 (d, 2H, Ar(C_2_)H and Ar(C_6_)H), 7.61 (d, 1H, Ar(C_2_)H, Ar(C_5_)H or Ar(C_6_)H), 8.32 (s, 1H, 5-NO_2_-imidazole-(C_4_)H), 9.28 (s, 1H, *N*-OH-imidazole-N-OH): ^13^C-NMR (125 MHz, DMSO-d_6_) δ 34.46 (5-NO_2_-imidazole-*N*-CH_3_), 56.41 (Ar(C_4’_)OCH_3_), 113.63 (Ar(C_2’_)H, 114.73 (*N*-OH-imidazole-(C_4_)), 116.10 (Ar(C_6’_)H, 118.21 (Ar(C_1’_), 125.91 (Ar(C_2_)H and Ar(C_6_)H), 127.46 (Ar(C_4_)), 128.48 (Ar(C_3_)H and Ar(C_5_)H), 132.70 (5-NO_2_-imidazole-(C_4_)H), 137.11 (Ar(C_1_)), 133.15 (*N*-OH-imidazole-(C_2_)), 136.53 (Ar(C_5’_)NO_2_, 137.02 (Ar(C_1_)), 139.74 (5-NO_2_-imidazole-(C_2_)), 140.09 (Ar(C_4’_)OH, 141.59 (5-NO_2_-imidazole-(C_5_)NO_2_), 144.67 (*N*-OH-imidazole-(C_5_)), 149.82 (Ar(C_3’_)OCH_3_: IR (KBr) ν_max_ cm^−1^: 3368 (ν *N*-O-H), 3130–3056 (ν C-H(aromatic), 1533–1503 (ν C=C(aromatic), 1369 (ν N=O_2_), 827 (ν C-N (Ar-NO_2_),: MS (ESI) *m/z*: 406.4 (100%). Anal. Calcd. for C_20_H_17_N_5_O_5_: C: 58.97; H: 4.21; N: 17.19. Found: C: 58.98; H: 4.21; N: 17.19.

*2'-(3-Hydroxy-4-methoxy-5-nitrophenyl)-1-methyl-5-nitro-5'-phenyl-1H,3'H-2,4'-biimidazol-3'-ol* (**12**). Red solid; mp 245–247 °C; yield 43%; ^1^H-NMR (400 MHz, DMSO-d_6_) δ 3.69 (s, 3H, 5-NO_2_-imidazole-*N*-CH_3_), 3.90 (s, 3H, Ar(C_3’_)OCH_3_), 7.25 (t, 1H, Ar(C_4’_)OH), 7.32 (t, 2H, Ar(C_3_)H and Ar(C_5_)H), 7.54 (d, 2H, Ar(C_2_)H and Ar(C_6_)H), 7.89 (s, 1H, 5-NO_2_-imidazole-(C_4_)H), 8.26 (d, 2H, Ar(C_2’_)H and Ar(C_6’_)H): ^13^C-NMR (100 MHz, DMSO-d_6_) δ 34.43 (5-NO_2_-imidazole-*N*-CH_3_), 55.41 (Ar(C_3’_)OCH_3_), 125.90 (Ar(C_2_)H and Ar(C_6_)H), 127.46 (Ar(C_4_)H), 128.44 (Ar(C_3_)H and Ar(C_5_)H), 132.70 (5-NO_2_-imidazole-(C_4_)H), 137.15 (Ar(C_1_)), 139.74 (5-NO_2_-imidazole-(C_2_)), 140.09 (5-NO_2_-imidazole-(C_5_)NO_2_), 141.59 (Ar(C_1’_)), 149.82 (Ar(C_3’_)OCH_3_): IR (KBr) ν_max_ cm^−1^: 3530 (ν O-H), 1555–1542 (ν C=C(aromatic), 1365 (ν N=O_2_), 827 (ν C-N (Ar-NO_2_),: MS (ESI) *m/z*: 451.4 (100%). Anal. Calcd. for C_20_H_16_N_6_O_7_: C: 53.10; H: 3.56; N: 18.58. Found: C: 52.98; H: 3.57; N: 18.55.

### 3.5. Activity against Bloodstream Trypomastigote Forms [28]

Stock solutions of NAH derivatives were prepared in DMSO. Bloodstream trypomastigote forms of *T. cruzi* (Y strain) were isolated from infected Swiss mice and re-suspended with Dulbecco’s modified Eagle medium plus 10% foetal calf serum (DMES) to a parasite concentration of 10 × 10^6^ cells/mL. This suspension (100 μL) was added to the same volume of each NAH derivative, previously prepared at twice the desired concentrations in DMES. Stock solutions of the NAH derivatives were prepared in dimethylsulfoxide (DMSO), and they were assayed in the range of 0.5 to 2000 μg/mL. The final concentration of DMSO never exceeded 0.5%, thus, it had no deleterious effect on the parasites [29].

### 3.6. Cytotoxic Effect on Murine Macrophages

Cellular viability in the presence and absence of TAI derivatives (**4**–**6**) was determined using Alamar Blue assay (Invitrogen). The macrophage cell line J774 was seeded into black clear flat-bottomed 96-well plates in a density of 2.5 × 10^6^ cells/well. After 1 h of incubation in controlled atmosphere (5% CO_2_, 37 °C),cells received fresh medium with or without Tween 20 (3%), DMSO (0.5%) and the TAI compounds (1 to 100 μM) in a quadruplicate assay. After 21 h of incubation in the previous conditions, 20 μL of Alamar Blue solution was added to each well and after 3 h, fluorescence was measured using SpectraMax M5/M5^e^ microplate reader (Molecular Devices; λ_exc_ = 555 nm, λ_em_ = 585 nm).

## 4. Conclusions

We have described herein a novel structural triarylheterocyclic template capable of displaying a significant antitrypanosomal profile *in vitro*. Among these *N*-hydroxytriarylimidazole (TAI) derivatives, we were able to identify the derivative 2'-(4-bromophenyl)-1-methyl-5'-phenyl-1*H*,3'*H*-2,4'-biimidazol-3'-ol (**6**), which showed moderate trypanocidal activity (IC_50_ = 23.9 µM) when compared to benznidazole, which is used as the standard drug. This compound did not present cytotoxic effects at concentrations near the IC_50_, being considered a good starting point in the development of new anti-Chagas drug candidates.
